# The Benefit of Detecting Reduced Intracellular B12 Activity through Newborn Screening Remains Unclear

**DOI:** 10.3390/ijns10020040

**Published:** 2024-06-18

**Authors:** Stella Knöpfli, Bernadette Goeschl, Maximilian Zeyda, Anna Baghdasaryan, Margot Baumgartner-Kaut, Matthias R. Baumgartner, Marion Herle, Julian Margreitter, Martin Poms, Saskia B. Wortmann, Vassiliki Konstantopoulou, Martina Huemer

**Affiliations:** 1 Division of Metabolism and Children’s Research Center, University Children’s Hospital of Zurich, University of Zurich, 8032 Zurich, Switzerland; stella.knoepfli@kispi.uzh.ch (S.K.);; 2 Department of Pediatrics and Adolescent Medicine, Division of Pediatric Pulmonology, Allergology and Endocrinology, Austrian Newborn Screening, Medical University of Vienna, 1090 Vienna, Austria; 3 Division of General Pediatrics, Department of Pediatrics and Adolescent Medicine, Medical University of Graz, 8036 Graz, Austria; 4 Newborn Screening Switzerland, Division of Clinical Chemistry and Biochemistry, University Children’s Hospital Zurich, University of Zurich, 8032 Zurich, Switzerland; 5 Department of Child and Adolescent Health, Division of Pediatrics I—Inherited Metabolic Disorders, Medical University of Innsbruck, 6020 Innsbruck, Austria; 6 Division of Clinical Chemistry and Biochemistry, University Children’s Hospital of Zurich, University of Zurich, 8032 Zurich, Switzerland; 7 University Children’s Hospital, Salzburger Landeskliniken and Paracelsus Medical University, 5020 Salzburg, Austria; 8 Department of Pediatrics, LKH Bregenz, 6900 Bregenz, Austria; 9 Vorarlberg University of Applied Sciences, Competence Area Healthcare and Nursing, 6850 Dornbirn, Austria

**Keywords:** cobalamin, breastfeeding, methylmalonic acid, homocysteine

## Abstract

Vitamin B12 (B12) deficiency (B12D) can have detrimental effects on early growth and development. The Austrian newborn screening (NBS) program targets inborn errors of cobalamin metabolism and also detects B12D. Of 59 included neonates with B12D suspected by NBS, B12D was not further investigated in 16 (27%) retrospectively identified cases, not confirmed in 28 (48%), and confirmed in 15 (25%) cases. NBS and recall biomarkers were recorded. Age at sampling of the dried blood spots for NBS and the 1st-tier methionine/phenylalanine ratio were the strongest parameters to predict B12D (67.4% correct allocations). No differences between cases with confirmed, unconfirmed, or unknown B12D or differences to norms were observed for growth and psychomotor development (Vineland III scales, phone interviews with parents of children between months 10 and 14 of life). B12 intake was below recommendations in most mothers. NBS can detect reduced intracellular B12 activity. No advantage of NBS detection and treatment regarding infant cognitive development or growth could be proven. Since conspicuous NBS findings cannot be ignored, and to prevent exposing newborns to invasive diagnostics, assessment of maternal B12 status during pregnancy seems advisable.

## 1. Introduction

Vitamin B12 (B12; synonym: cobalamin) is essential for infant growth and development, and early B12 deficiency (B12D) can have detrimental effects. Meat and dairy are the primary dietary sources of B12 [1,2,3]. Holotranscobalamin (Holo-TC) is the active form of B12 that is able to enter cells. Intracellularly, B12 is essential for the remethylation of the amino acid methionine (Met) from homocysteine (Hcy) by Met-synthase in the cytosol, as well as the formation of succinyl-CoA from methylmalonyl-CoA by methylmalonyl-CoA mutase in the mitochondrial matrix. The remethylation reaction from Hcy to Met provides methyl groups that are essential for numerous reactions, such as nucleotide, neurotransmitter, and myelin synthesis, as well as DNA and RNA methylation [4].

Since 2018, the newborn screening (NBS) program in Austria has targeted inborn errors of B12 metabolism (e.g., the cobalamin C defect) using an algorithm that also identifies neonates that may have B12D. Neonatal B12D is usually mediated by the maternal B12 status [5,6]. Maternal B12D can be caused by pathogenic variants in genes coding for proteins essential for B12 absorption and trafficking; by acquired conditions impairing B12 absorption, such as antibodies against intrinsic factors or gastrointestinal surgery; or by insufficient consumption of foods from animal sources (e.g., in vegans) [3].

Neonates identified by the Austrian NBS are obligatorily recalled to investigate them for inborn errors of B12 metabolism, organic acidurias, and B12D. For selective diagnostics, urine and dried blood spots (DBS) are collected, and venous blood samples are taken to measure ammonia, blood gases, organic acids, B12, methylmalonic acid (MMA), total Hcy (tHcy), Met, and holo-TC [5].

Since NBS recalls are generally stressful situations for families [7], and, presently, no universally accepted definition of meaningful neonatal B12D and its potential consequences during infancy is in place, the optimal management of suspected neonatal B12D is under discussion [8,9].

Severe, long-standing infant B12D can cause severe clinical symptoms such as irritability, failure to feed and thrive, apathy, developmental delay, seizures, and cerebral atrophy in the first year of life. Affordable, successful treatment with B12 is easily available [10]. The identification of newborns at risk of developing such a severe course would be a main benefit delivered by NBS. Unfortunately, most infants with clinically diagnosed severe B12D show normal values of the B12-related parameters tested in NBS algorithms as neonates, and the condition remains undetected [9,11]. Making B12D a primary NBS target has also been suggested to detect neonates with only suboptimal B12 status [8,12,13,14], since lower B12 values have been shown to correlate with less genomic stability of human cells [15] and poorer mental performance in infants in some reports [16].

In contrast to Austria, neonates with suboptimal or deficient B12 status remain undetected in Switzerland, where B12-associated diseases are not part of the screening panel.

This study investigates whether children who are suspected to have B12D by NBS have a benefit outweighing the risks and burdens of detection. A retrospectively identified cohort of Swiss children was investigated to address the question of whether a cohort with 1st- and 2nd-tier positive screening, but without further workup, would have a disadvantage in terms of neurocognitive development. For the regular Austrian NBS cohort, the positive predictive value (PPV) of the NBS algorithm for B12D was assessed, and biochemical parameters, maternal and child nutrition, and psychomotor development and physical growth between months 10 and 14 of life were studied. Comparisons were drawn between children with normal and deficient B12 statuses detected by NBS at the recall investigations.

## 2. Materials and Methods

Neonates were identified as having suspected B12D either during the regular course of the Austrian NBS or retrospectively by re-analyzing DBS collected by the Swiss Newborn Screening Program between 1 September 2020 and 30 September 2021 and between 1 January 2022 and 28 February 2022. Although not targeted by the Swiss NBS program, tandem mass spectrometry generates B12-specific biochemical parameters. The Austrian first-tier test criteria were applied to identify B12D in Switzerland. Since mass spectrometric measurement results vary significantly between laboratories, cut-offs are generally based on percentiles to allow for comparability. First-tier testing was considered positive if (a) propionylcarnitine (C3) exceeded the 99th percentile (pt); (b) Met was lower than the 0.2nd pt; (c) the Met/Phenylalanine (Phe) ratio was lower than the 0.2nd pt; or (d) C3 exceeded the 95th pt and at least one of the following conditions also applied: C3/Met > 99.5th pt; C3/palmitoylcarnitine (C16) > 99.5th pt; C3/acetylcarnitine (C2) > 99.8th pt; and C3/free carnitine (C0) > 99.5th pt.

In Austrian children, second-tier testing was conducted as part of the NBS routine, as described above. THcy concentrations > 12 µmol/L in the first DBS triggered an immediate recall, and if tHcy concentrations exceeded 10 µmol/L in the first DBS, a second DBS was requested. If tHcy exceeded 12 µmol/L in the second DBS, an immediate recall for further diagnostics followed. At the recall visit, B12D was confirmed by serum vitamin B12 < 150 pmol/L, holoTC < 25 pmol/L, MMA > 600 nmol/L in serum, or due to being detectable in urine. Children meeting these criteria were treated with at least one intramuscular injection of 1 mg hydroxocobalamin.

First-tier identified Swiss DBS were labeled using a unique numerical code without identifying information and shipped to the Austrian NBS Program at University Children’s Hospital Vienna for measurement of the second-tier parameter tHcy. Due to the retrospective identification, second DBS cards were unavailable for Swiss newborns with tHcy values between 10 and 12 µmol/L in the first DBS, and the tHcy measurements for study purposes were performed using DBS which had been stored for several months. Therefore, the tHcy cut-off in the DBS of Swiss neonates was adjusted to the 96th pt of measured samples. The codes of DBS with positive first- and second-tier results were sent to the Swiss newborn screening program at University Children’s Hospital of Zürich and decoded for the recruitment process.

Term infants (38–42 weeks gestation) identified as B12D either by the regular Austrian NBS B12D_NBS_A) or retrospectively in Switzerland (B12D_NBS_CH) were eligible for participation. Parents of children born and screened in Austria between 1 November 2019 and 31 March 2022 were invited for participation. In Austria, the parents’ contact details are given on the DBS card, which allowed the study team to contact them directly. In Switzerland, the contact data of the birth hospital or midwife, and, less often, the parents’ contact data, are given on the DBS card. Either birth clinics or midwives were asked to inform the parents about the study, or parents were contacted directly. After obtaining informed parental consent, an appointment for a phone interview when the child was between 10 and 14 months old was made if no major language barrier made participation in the interview impossible.

**Figure 1:**
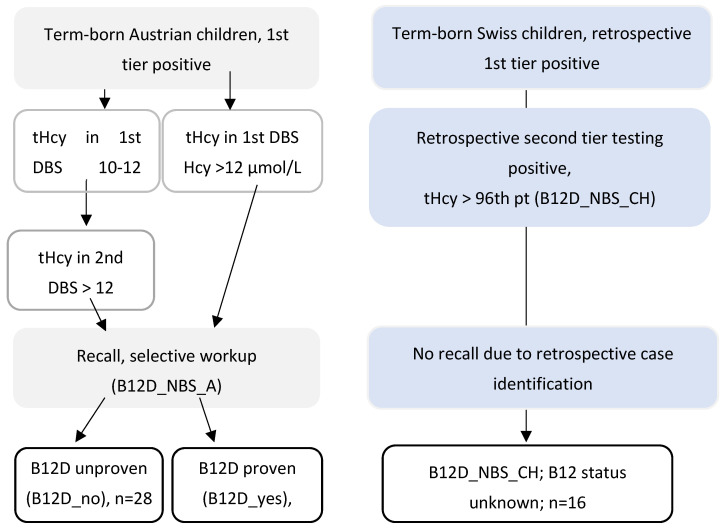
Study algorithm and groups.

Since Swiss children were not subjected to B12-related diagnostic workup, their B12 status remained unknown. B12D_NBS_A cases were divided into subgroups with normal B12-related diagnostic workup (B12D_no) and proven and treated B12D (B12D_yes) (Figure 1).

Biochemical data from NBS and recall workup were retrieved, and parents participated in a 45 min phone interview conducted by a specifically educated member of the study staff when their children were between 10 and 14 months old. Information on the infant’s present auxological data and feeding history, medical problems, and requirements for early intervention or functional therapies, as well as maternal nutritional habits, socio-demographic data, social circumstances, and language/s spoken at home, were obtained. Maternal B12 intake during pregnancy (from food and/or supplements) was estimated based on the questionnaire data and categorized as being within or below the recommended dietary allowances. In the second part of the interview, the Vineland Adaptive Behavior Scales, Third Edition (Vineland III) were applied to assess the development of the children. The normative values of the Vineland III scales were used, which contain month-by-month standardization and allow for age-at-test-adapted evaluation. Biochemical (NBS, recall) and interview data were entered into a safe database (RedCap) based at the University Children’s Hospital Zurich.

### Statistical Analysis and Power Calculation

The Vineland scales are a standardized procedure with a mean of 100 and a standard deviation (SD) of 15 (values < 85 are considered below average). The study intended to find differences exceeding 1 SD between the mean scores of Austrian children identified as screening positive and the retrospectively detected Swiss children. The effect size for this difference of means would be 1 (large effect from 0.8 according to Cohen), and the number of subjects required to find such a difference would be 48 (with d = 1, α = 0.05, 1 − ß = 0.90).

Descriptive analysis of the study parameters was performed for the entire cohort (B12D_NBS), as well as for the subgroups (B12D_NBS_A and B12D_NBS_CH). Biochemical parameters were compared between the B12D_yes and B12D_no groups. Stepwise discriminant analysis and stepwise regression analyses of the B12D_NBS_A cohort were performed to investigate whether B12_D status at recall could be predicted from the NBS results, and correlations between the NBS parameters, time of first DBS sample collection, and recall markers were calculated. The B12D_NBS_A cohort was divided into mothers with reported B12 uptake within the recommended dietary allowances of 4.5 µg/d during pregnancy and mothers with lower B12 intake. Subsequently, infant biomarkers were compared between the two groups. For group comparisons of continuous variables, independent *t*-tests or Mann–Whitney-tests (if data were not normally distributed) were utilized. For categorical data, Fisher’s exact test or the Fisher–Freemann–Halton test were applied. To investigate relationships between variables, Pearson or Spearman correlations were calculated depending on the distribution of the data. All calculations were performed with SPSS (IBM SPSS Statistics 25.0) and Graphpad. *p*-values ≤  0.05 (all two-tailed) were considered statistically significant. Bonferroni–Holm correction for multiple comparisons was only applied to group comparisons of the Vineland III development scores.

## 3. Results

### 3.1. The Positive Predictive Value of the Austrian NBS Algorithm for B12D

Of the 203.440 neonates screened in Austria between 1 November 2019 and 31 March 2022, 7.127 (3.5%) children had a positive first-tier test, and the second-tier parameter tHcy was assessed. In addition, 161 (2.3%) of them were also second-tier positive. Following the routine of the Austrian NBS, a clinical recall for selective diagnostics of the B12 status was initiated. A total of 106 children had no evidence of B12D. In 55 cases, B12D was proven according to the following criteria: serum B12 < 150 pmol/L or HoloTC < 25 pmol/L or MMA in serum > 600 nmol/L, resulting in a PPV of the screening algorithm of 34%.

### 3.2. Study Groups

For this study, data sets from 43 (27%; 23 males) Austrian children who screened as positive for B12D and were recalled were analyzed (B12D_NBS_A). The remaining 12 patients were recalled elsewhere, and their B12 statuses were unavailable. The mean gestational week at birth was 39.4; the median minute 1, 5, and 10 APGAR scores were 9, 10, and 10; the mean birth weight was 3465 ± 460 g, and the median head circumference was 35 cm (interquartile range: 2). At recall workup, 28 children had no evidence of B12D (B12D_no), while 15 proved to be deficient (B12D_yes).

In Switzerland, 114.480 neonates were screened during the study period. Of these, 3362 (2.9%) children were first-tier, and 140 (4.2%) were first- and second-tier positive; second-tier testing was not possible for technical reasons in 271 cases (8.1%). Of the 140 identified children, the parents could not be contacted in 48 cases, did not give their consent in 14 cases, and 62 children had to be excluded due to premature birth or language barriers of the parents, making the study interview impossible. Study interviews were conducted in the remaining 16 cases (9 males); biomarker data sets were complete for 14/16 cases. The mean gestational week at birth was 40.2; the median minute 1, 5, and 10 APGAR scores were 8, 9, and 10; the mean birth weight was 3337 ± 411 g; and the median head circumference was 35 cm (interquartile range: 1.9). Due to the retrospective identification of cases, none of the Swiss participants had a recall for B12 status workup.

### 3.3. Collection Time and Biomarker Data from NBS

The age at collection of the first DBS for the newborn screening was 2.4 (Md 2, range 1–4 days) for the entire B12D_NBS (*n* = 57) group, with a significantly later (*U* = 109.5; *p* < 0.001) collection time in Swiss compared to Austrian children (M 2.1; Md 2, range: 1–4 days).

DBS were collected significantly later, and Met and Met/Phe were significantly lower in the B12D_yes compared to the B12D_no group (*U* = 119; *p* = 0.01 and t = −2.16; *p* = 0.036 and t = −2.68; *p* = 0.01). Stepwise discriminant analyses revealed that the age at sampling of the first DBS and the Met/Phe ratio allocated 67.4% of the cases to the B12D_yes and B12D_no groups correctly (Wilks-Lambda = 0.78; Chi square 9.577; df = 2; *p* = 0.008; canonical correlation 0.476).

**Figure 2:**
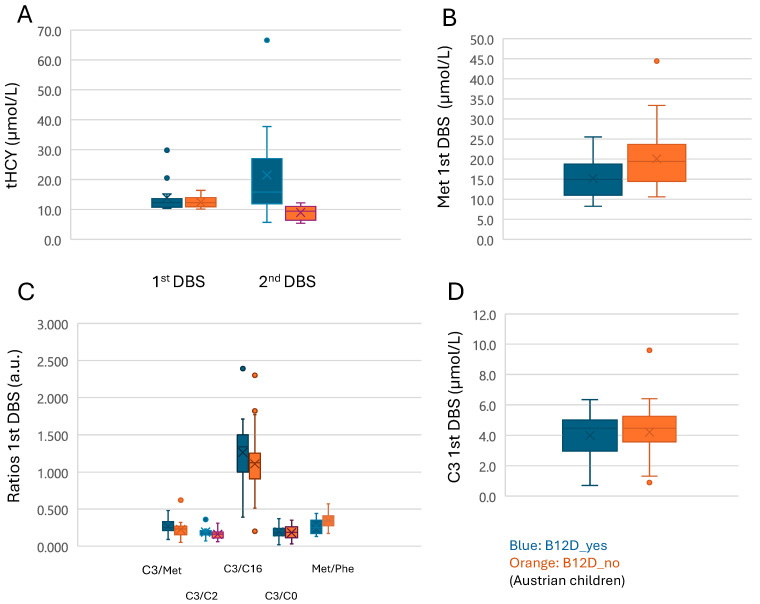
(**A**–**D**): First- and second-tier markers in the Austrian cohort (B12D_yes in blue, B12D_no in orange).

The first-tier parameters were analyzed for the entire B12D_NBS group and separately for children from Austria and Switzerland (Table 1). First- and second- tier markers in the Austrian cohort are shown in Figure 2. To account for the variability between laboratories for tHcy measurements, the relations between first- and second-tier testing were analyzed separately for Austrian and Swiss cases. Beyond the expected correlations between biomarkers and their related ratios (*p* < 0.001 for C3 and C3/C2, C3/C16, C3/C0; Met and Met/Phe), Pearson correlations between Met and C3 reached significance for the B12D_NBS group (r = 0.328; *p* = 0.013) and the subgroup of Austrian neonates (r = 0.397; *p* = 0.008). The C3/C0 ratio correlated significantly with tHcy in the Austrian (ρ = 0.416; *p* = 0.006) and in the Swiss (r = 0.598; *p* = 0.024) neonates; the mean tHcy was 7.4 (median: 7.1; range: 5.3–11.4).

### 3.4. Analysis of Collection Time and Biomarkers from NBS and at Recall in Austrian Children

Mean (median, range) of the second-tier parameter tHcy and of the recall parameters in Austrian neonates are shown in Table 2 and Figure 2. Correlations between NBS parameters, time of first DBS sample collection, and recall markers were analyzed in 22 Austrian children (B12D_yes =15; B12D_no = 7) with complete data sets. The age at first DBS sampling correlated significantly with the recall parameters tHcy (*n* = 19; ρ = 0.625; *p* = 0.004), serum MMA (*n* = 16; ρ = 0.523; *p* = 0.038), and inversely with holo-TC (*n* = 19; ρ = −0.483; *p* = 0.036).

C3/C2 in the first DBS correlated significantly with MMA at recall (*n* = 16; r = 0.594; *p* = 0.015).

Stepwise regression analyses revealed that C3/C2 in the first DBS predicted serum and urinary MMA at recall (*n* = 15; r = 0.59; F (1, 13) = 6.776; *p* = 0.022; R^2^_corr_ = 0.292; and *n* = 17; r = 0.54; F (1, 15) = 6.173; *p* = 0.025; R^2^_corr_ = 0.244). Second-tier tHcy and age at first DBS sampling significantly predicted recall tHcy in DBS and in plasma (*n* = 19; r = 0.7 and r = 0.378; F (2, 16) =14.417; *p* < 0.001, R^2^_corr_ = 0.599; and (*n* = 16; r = 0.68 and r = 0.56; F (2, 13) = 22.630; *p* < 0.001; R^2^_corr_ = 0.743).

Age at initial DBS sampling also predicted holo-TC at recall (*n* = 18; r = −0.68; F (1, 16) = 13.388; *p* = 0.002; R^2^_corr_ = 0.422). B12 concentrations at recall were not predicted by any of the NBS parameters.

At recall, B12 was <150 pmol/L in 12 cases, MMA in urine was detected in 13 and elevated > 600 nmol/L was detected in serum in 10 cases, and holo-TC was <25 pmol/L in 11 children.

### 3.5. Maternal and Infant B12 Supply and Their Impact on Biomarkers

As a result, 25% of mothers in the B12D_NBS_CH group, 13% in the B12D_NBS_A_yes group, and 21% in the B12D_NBS_A_no group reported to follow a vegan or vegetarian diet (no significant between-group differences). Of Swiss and Austrian mothers, 44% took variably dosed supplements containing unspecified amounts of B12 during pregnancy, but the estimated maternal B12 intake from food and supplements was within the recommended dietary allowances of 4.5 µg/d during pregnancy in only 21 of 55 (38%) cases [17].

Insufficient estimated maternal B12 intake was associated with child serum MMA at recall (ρ = −0.521, *p* = 0.038) but not with other infant biomarkers, probably owing to the small sample size and the rough estimate of B12 intake by retrospective questioning.

Exclusive breastfeeding from birth until the introduction of solid foods at a median age of 5 months was significantly more frequent in the B12D_yes group (14 cases; 93.3%) compared to 50% in the B12D-no group (*p* = 0.006). At time of the interview, 2 children were on a vegan diet, 7 children were on a vegetarian diet, and 50 were on a mixed diet. Eleven infants (73.3%) in the B12D_yes group were still partially breastfed compared to twelve (42.9%) children the B12D_no group. The B12 concentrations were lower in initially breastfed children compared to formula-fed infants (all differences not significant).

Maternal body mass index and socioeconomic family background had no significant impact on B12 status and did not differ significantly between the B12D_NBS subgroups from Austria and Switzerland or between Austrian B12D_yes and B12D_no children at the age of 10 to 14 months.

### 3.6. Growth and Development Data

The B12D_NBS group and the subgroups were analyzed for clinical signs and symptoms of B12D. Standard deviation scores for weight and height at time of the interview, as well as a comparison of percentiles between the measurements at the interview time and at birth, showed no evidence of failure to thrive. The scores for communication (receptive and expressive), daily living skills, socialization, motor skills (gross and fine motor development), and the adaptive behavior composite that were established using an interview based on the Vineland III scales showed no significant between-group differences (Swiss versus Austrian children, B12D_yes versus B12D_no groups). None of the results from any of the groups showed significant deviations from the norms of the instrument that were established in a population from the US.

However, in the Austrian cohort, five (33.3%) B12D_yes and seven (25.9%) B12D_no cases scored below average (mean-1 SD) on the gross motor scale. The five children with below-average gross motor skills in group B12D_yes were recalled (and treated) significantly later (mean 53 (54 days)) than B12D_yes children with normal gross motor scores (mean 26 (35 days)); (*p* < 0.001 and (*p* = 0.007)).

## 4. Discussion

The algorithms of NBS programs that target inborn errors of B12 metabolism also identify neonates that may have B12D [4,6,18]. It is currently discussed whether the identification of neonates who may have B12D is a potentially valuable byproduct of NBS or whether B12D should even become a primary target of NBS programs [14].

In 34% of the Austrian children suspected by NBS to have B12D in this study, the condition was proven. This PPV of the Austrian algorithm is lower than published before [5], but exceeds, e.g., the PPV for cystic fibrosis [19]. Our results suggest that special consideration of Met and the Met/Phe ratio, as well as the time of DBS sampling, may improve the detection ratio towards B12D cases proven at recall. While NBS to detect B12D thus seems technically feasible, the confirmation and definition of B12D in neonates are still under discussion. Usually, the definition of B12D is based on B12, holoTC, MMA, and tHcy measurements at recall. It is known that common B12 measurements in the blood reflect the circulating, mostly haptocorrin-bound fraction of B12 that does not necessarily equal the intracellularly active B12 fraction, holo-TC, for which age-specific norms for neonates have not been established so far [4]. B12 in blood is considered normal at >250 pmol/L, low at between 150 and 249 pmol/L, and deficient at below 150 pmol/L [20,21,22]. The cutoff value of <150 pmol/L has been established based on the observation of clinical signs of B12D at such concentrations [22]. Yet, in vitro studies in human cells show that B12 concentrations need to be as high as >300 pmol/L (and plasma homocysteine < 7.5 μmol/L) to prevent genomic instability [15], and it has been suggested that even higher B12 values be considered as deficient, particularly if they are associated with elevated tHcy [23].

Recently, 95% reference intervals for B12 (180–1400 pmol/L) have been published for infants in their first year of life based on data from Denmark and Canada [24,25]. While reference intervals are useful to depict the distribution of B12 blood concentrations in a population with specific nutritional, genetic, and lifestyle backgrounds, they may be less decision-supportive for specific cases and clinical situations [20].

THcy and MMA increase when serum B12 is low [18], and in the Austrian recall algorithm, the diagnosis of functionally relevant B12D is supported by elevated tHcy or MMA concentrations. The current cutoff of the algorithm for tHcy has been defined as 12 µmol/L, but this value is not undoubted. Cutoffs of 6.5 or 8 µmol/L tHcy have been suggested [26,27], and better neurocognitive development of infants with lower tHcy has been reported [26,27]. The cut-off of 6.5 µmol/L tHcy was derived from a cohort study in children who were supplemented with B12 at 6 weeks and reevaluated at month four. THcy of 6.5 µmol/L corresponded to the 97.5 percentile in supplemented children [23]. THcy of 6.5 µmol/L corresponded to the 70th percentile in the Austrian cohort, meaning that the use of 6.5 µmol/L as the cut-off would result in a recall rate of nearly 1/3 of first-tier-positive neonates. Such an approach would challenge the requirements for newborn screening programs to be feasible, acceptable for the public, and financially sustainable. Newborn screening, thus, seems not to be the method of choice to optimize B12 status [23] in neonates.

Elevated MMA in the blood or urine is a valuable additional marker of functional B12D [4], but has been suggested to be less so in early in life, since MMA is derived from odd-chain fatty acids that are particularly high in breastmilk and generally higher in breastfed infants [23,28]

A pronounced increase in tHcy and MMA was observed when B12 decreased below 250 to 275 pmol/L [21], suggesting that MMA and tHcy are useful in detecting cases of impaired intracellular B12 activity despite B12 being in the low, but not deficient, range [22,24,25]. In our population, MMA and tHcy at recall correlated significantly with NBS markers, while B12 concentrations did not, confirming that B12 is an unreliable marker for impaired intracellular B12 function, at least at this age.

This study has several limitations. Children from Switzerland were screened retrospectively, and their second-tier tHcy was based on percentiles and not on the numeric cutoffs used in the Austrian algorithm. Furthermore, systematic variability of biomarkers due to assessment in two laboratories must be expected. The neonatal B12 status remained undetermined in the Swiss children; thus, the distribution of children with and without proven B12D in this group cannot be quantified. This impairs the significance of the finding that this group achieved without differences in norms regarding cognition and physical growth. Austrian children were recalled and subjected to selective B12D workup in a broad time frame. The sample sizes of the study are small, mostly due to obstacles such as irretrievable or invalid contact information, language barriers, refusal of study participation by the parents, or missing data. The low percentage of participants from the Swiss first- and second-tier-positive children (16/140) increases the probability that our sample may not be representative of the overall cohort. The parental interviews on the children’s neurodevelopment were carried out between month 10 and 14 of life, and the infants’ diets may have varied over this time span; this may have an impact on neurodevelopment. The use of a developmental test such as the Vineland III scales is the gold standard for the evaluation of child development. To include as many cases as possible, we decided in favor of a phone interview with the parents based on the Vineland III scales. We tested the comparability of this procedure with face-to-face neurodevelopmental testing (Bayley III, Griffiths Scales) in 53 children aged 10 to 14 months and found a substantial correlation between the overall scores of the Vineland III interview and traditional developmental testing (unpublished data). Our main aim was to investigate the benefit or clinical advantage for children detected as potentially B12D by NBS. It has been shown that symptomatic children who were clinically diagnosed with severe B12D in their first months of life were not suspected to have B12D by NBS algorithms (including the Austrian) retrospectively applied to dried blot spot cards collected in their first days of life [9,11]. This contradicts the assumption that NBS can prevent the classical picture of severe, symptomatic B12D and suggests that newborns detected as B12D by NBS represent a population with acute suboptimal intracellular B12 activity rather than a population prone to clinically symptomatic, severe infant B12D.

Whether the detection by NBS and treatment of suboptimal intracellular B12 activity prevents other, more subtle health problems and, thus, indicates added value against costs and burdens is not clear. Investigations of the relation of B12 status or intake of mothers and children with infant cognitive development and neurological conditions show variable results. THcy concentrations even slightly above normal correlated with reports of tremor, impaired fine-motor performance, and excessive sleeping in a cohort of 25 Norwegian children [27]. A meta-analysis of 17 studies showed more favorable cognitive performance of children to be associated with higher maternal or child B12 status or intake in 10 studies. No significant association was found in five studies, and a less favorable cognitive performance associated with higher maternal or child B12 status or intake was found in two studies [16]. The variability of the results can be partly explained by differences between studies in the interpretation of B12 concentrations, the definition and operationalization of outcome parameters to represent cognitive abilities, the different ages of the children at investigation, the numerous confounding predictors of cognitive performance, and the heterogeneity of the genetic and nutritional backgrounds of the reported populations [16]. While it is beyond doubt that B12 is essential for early development, the evidence that lower B12 is a precise enough single predictor for a less favorable cognitive development is weak. In the present study, the cognitive outcome was equal between groups, and none of the groups scored below the norms of the Vineland III scales. This is especially interesting regarding the group of Swiss children that probably included children with true, but undetected and untreated, B12D.

However, the finding of this study that below-average gross motor function in proven B12D cases correlates with the time to recall and treatment must be considered and followed up in a prospective observational trial.

Many factors determine B12 status in young children, mainly maternal B12 status during pregnancy and breastfeeding, prematurity, and low birth weight [29]. Furthermore, the use of nitrous oxide during labor irreversibly inhibits the function of the enzyme methionine synthase and significantly affects infant tHcy [30,31,32].

Although we cannot presently unequivocally prove or exclude that reduced intracellular B12 availability is associated with less favorable development and neurological symptoms [27], each B12-related positive NBS triggers thorough metabolic workup. Any NBS recall or metabolic workup means exposure of neonates to invasive procedures and has an impact on parents’ attitude towards their children’s health [7].

Observational studies show declining maternal B12 concentrations during pregnancy [33], and in our study, most women reported a B12 intake below the recommendation during pregnancy. In infants, B12 decreases and tHcy and MMA rise during the first months of life; infant B12 status is lowest between weeks 6 and 24 [34], with breastfed children having less B12 and higher tHcy and MMA compared to formula-fed children [18].

Maternal B12 status strongly predicts infant B12, MMA, and tHcy concentrations [18]. Not only is a maternal B12 concentration > 275 pmol/L in week 18 of pregnancy correlated with a sufficient infant B12 status in the first months of life [35], but maternal B12 status in the first trimester also predicts the infant MMA-related markers at NBS [33].

Therefore, screening of the maternal B12 status either by self-reporting of B12 intake or by measurement of relevant parameters during early pregnancy, followed by counseling and, if necessary, B12 supplementation, could probably reduce invasive and cost-intense B12D-related NBS recalls. NBS certainly remains helpful in detecting neonates of mothers who were not reached by the preventive strategy or who have impaired B12 status due to, e.g., nitrous oxide use during labor or maternal disorders of B12 uptake and processing.

## 5. Conclusions

B12 intake during pregnancy was reported to be lower than recommended by most mothers in this study. Maternal B12 status predicted infant NBS biomarkers. MMA and tHcy at recall correlated with NBS first- and second-tier markers, while B12 concentrations did not. NBS detected that reduced intracellular B12 activity has an impact on infant neurocognitive development that is not exactly quantifiable. Neurocognitive development of the study groups did not differ nor deviate from norms, but later treatment in proven B12D was associated with more gross motor scores below the norms. NBS recalls cause invasive diagnostics and exert strain on children and their families. Since maternal B12 status during pregnancy determines neonatal and infant B12 status, preventive interventions targeting maternal B12 status in early pregnancy should be considered to reduce B12-related recalls.

## Figures and Tables

**Table 1:** Mean (median, range) of the first-tier parameters per group and age at DBS collection.

	*n*	Age at DBS Collection (Days)	C3 µmol/L	C3/Met	C3/C2	C3/C16	C3/C0	Met µmol/L	Met/Phe
All cases: B12D_NBS_A and B12D_NBS_CH	57	2.4 (2; 1–4)	4.2 (4.4; 0.7–9.6)	0.25 (0.23; 0.05–0.68)	0.16 (0.16; 0.06–0.36)	1.15 (1.14; 0.2–2.4)	0.18 (0.18; 0.02–0.37)	18.1 (17.9; 7.4–44.4)	0.3 (0.29; 0.11–0.74)
B12D_NBS_CH	14	3.1 (3; 1–4)	4.2 (4.3; 1.75–7.2)	0.28 (0.23; 0.1–0.68)	0.13 (0.11; 0.07–0.26)	1.09 (1.08; 0.5–2.2)	0.18 (0.18; 0.07–0.3)	17.4 (18.4; 7.4–25.9)	0.26 (0.25; 0.11–0.74)
B12D_yes (A)	15	2.6 (3; 2–4)	4 (4.5; 0.7–6.3)	0.27 (0.25; 0.09–0.48)	0.19 (0.19; 0.07–0.36)	1.26 (1.34; 0.4–2.4)	0.19 (0.18; 0.02–0.37)	15.2 (14.9; 8.24–25.5)	0.26 (0.25; 0.13–0.44)
B12D_no (A)	28	2 (2; 1–3)	4.2 (4.5; 0.9–9.6)	0.22 (0.23; 0.05–0.62)	0.16 (0.16; 0.06–0.31)	1.11 (1.12; 0.2–2.3)	0.18 (0.19; 0.03–0.35)	20 (19.4; 10.6–44.4)	0.35 (0.34; 0.17–0.57)

**Table 2:** Mean (median, range) of the second-tier parameter tHcy and of the recall parameters in Austrian neonates.

	tHcy µmol/L Second-Tier Test in 1st DBS	tHcy µmol/L at Recall (DBS)	tHcy µmol/L at Recall (Plasma)	MMA mmol/mol Creatinine at Recall (Urine)	MMA nmol/L at Recall (Serum)	Holo-TC pmol/L at Recall	B12 pmol/L at Recall
B12D_NBS_A	N = 43 12.9 (12.3; 10.1–29.8)	N = 19 19.9 (12.3; 5.4–66.6)	N = 17 22.6 (19.5, 4.8–54.0)	N = 18 46.2 (8.5; 0–262)	N = 16 4539.3 (1198.0; 190–25,242)	N = 19 25.39 (17.0; 5.5–96.1)	N = 22 149.5 (111.0; 74.0–450.0)
B12D_yes (A)	N = 14 13.9 (12.3; 10.3–29.8)	N = 12 24.5 (18.0; 5.7–66.6)	N = 11 28.3 (24.0; 10.7–54.0)	N = 13 58.2 (10.0; 1–262)	N = 12 5874.3 (3647.5; 455–25,242)	N = 13 16.5 (13.8; 5.5–41.0)	N = 15 109.3 (96.0; 74–228)
B12D_no (A)	N = 28 12.4 (12.3; 10.1–16.4)	N = 7 12.0 (10.8; 5.4–29.0)	N = 6 12.0 (10.6; 4.8–23.0)	N = 5 14.8 (2.0; 0–63)	N = 4 534.3 (523.5; 190–900)	N = 6 44.7 (39.2; 15.0–96.1)	N = 7 235.6 (205.0; 140–450)

## Data Availability

Access to the data included in the analyses can be provided upon request to the corresponding author.
